# Two new species of *Euglossa* from South America, with notes on their taxonomic affinities (Hymenoptera, Apidae)

**DOI:** 10.3897/zookeys.221.3659

**Published:** 2012-09-13

**Authors:** Ismael A. Hinojosa-Díaz, André Nemésio, Michael S. Engel

**Affiliations:** 1 Division of Entomology, Natural History Museum, and Department of Ecology & Evolutionary Biology, University of Kansas, 1501 Crestline Drive – Suite 140, University of Kansas, Lawrence, Kansas 66045, USA; 2 Current address: Department of Environmental Studies, Emory University, Math & Science Center – Suite E536, 400 Dowman Drive, Atlanta, Georgia 30322, USA; 3 Instituto de Biologia, Universidade Federal de Uberlândia. Rua Ceará, s/n, Campus Umuarama, Uberlândia, MG, 38.400-902, Brazil

**Keywords:** Orchid bees, *Glossurella*, taxonomy, Atlantic Forest, Brazil, Colombia

## Abstract

Two new species of the genus *Euglossa* Latreille, subgenus *Glossurella* Dressler are here presented. *Euglossa (Glossurella) embera* **sp. n.**, from the Pacific lowlands of Colombia, and *Euglossa (Glossurella) adiastola* **sp. n.**, from the Atlantic Forest of Brazil. Their taxonomic association and distinction are discussed, as well as the correct understanding of the subgenus *Glossurella*.

## Introduction

The appealing external morphology and interesting behavioral features of orchid bees make them one of the most notorious groups among the Neotropical bee fauna. Males of this group of bees have a characteristic set of secondary sexual morphological features involved in the collection of aromatic substances from floral and non-floral resources, notably from flowers of Orchidaceae (Dressler 1982a). The aromatic compounds are exposed during mating (Eltz et al. 2005). Of the five genera comprising the group, *Euglossa* Latreille is the most diverse with around 130 species (Nemésio and Rasmussen 2011). Since the discovery of the chemicals involved in the attraction of euglossine males to orchid flowers (Dodson et al. 1969), numerous new species have been described. Revision of historical material, discovery of new suites of morphological features, and access to newly collected sets of specimens in recent years has contributed to the recognition of various new species, particularly in *Euglossa* (e.g., Hinojosa-Díaz and Engel 2007, 2011a, b; Hinojosa-Díaz et al. 2011; Nemésio 2007, 2011b, c; Parra et al. 2006; Ramírez 2005, 2006; Rasmussen and Skov 2006). Here we describe and illustrate two new species of *Euglossa* in the subgenus *Glossurella* Dressler, one from the Pacific lowlands of Colombia and another from the Atlantic Forest of Brazil. We discuss the taxonomic affiliation of both species and one possible re-interpretation of the subgenus *Glossurella* in the light of current phylogenetic hypotheses.

## Material and methods

Specimens here examined belong to the following institutions: Division of Entomology, University of Kansas Natural History Museum, Lawrence, Kansas, USA (SEMC), and the Entomological Collection, Universidade Federal de Minas Gerais, Belo Horizonte, Minas Gerais, Brazil (UFMG). Label information for each specimen is presented enclosed by quotation marks (“”), each label separated by double slash symbols (//), and every row on individual labels separated by a semicolon in italics (;), all of this followed by the number and sex of individuals corresponding to that dataset.

Morphological terminology in general follows that of Engel (2001), Michener (2007), and Hinojosa-Díaz (2008); some procedures for establishing metrics follow those of Brooks (1988). Species descriptions follow the overall format for other *Euglossa* species as presented by Hinojosa-Díaz and Engel (2007, 2011a, b) and Hinojosa-Díaz et al. (2011). Photomicrographs were prepared using a Cannon EOS 7D digital camera and an Infinity K-2 long-distance microscope lens. Multilayer images were produced by using the software CombineZP.

## Systematics

### Euglossa (Glossurella) embera sp. n.

urn:lsid:zoobank.org:act:8FC01C8B-6EA5-469D-BAEF-8F258B5EB93B

http://species-id.net/wiki/Euglossa_embera

**Figures 1–2.**
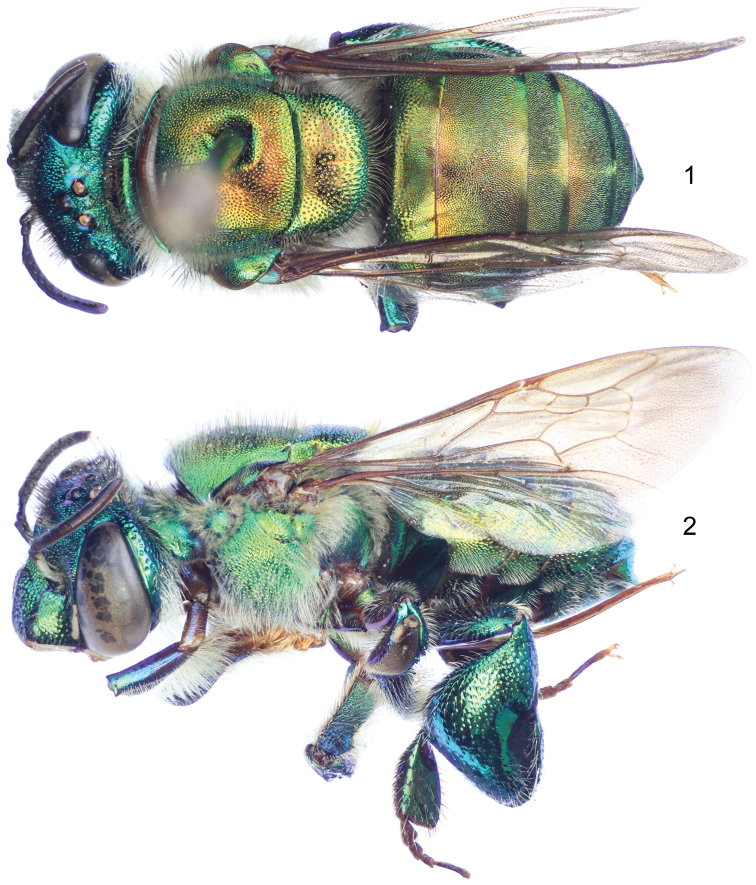
*Euglossa embera* sp. n., male holotype. **1** Dorsal habitus **2** Lateral habitus.

Figs 1 [Fig F2] [Fig F3] 20

#### Holotype.

♂, labeled: “COLOMBIA: Prov. Valle; Rio Anchicaya, 400m.; 10 Feb. 1977. M.D.;Breed & C.D.Michener”. The holotype is in SEMC.

#### Paratypes.

3♂♂, 1♀: labeled as follows: data as holotype (1♂); data as holotype plus two extra labels “2 // Euglossa; bursigera Moure; det. R.L.Dressler 1977” (1♂); label data as holotype except date and collectors “IX-28-76. Bell,; Breed & Michener” (1♂); label data as holotype except date “11 Feb. 1977” (1♀). Paratypes are deposited in SEMC and UFMG.

#### Diagnosis.

**Figures 3–4. F2:**
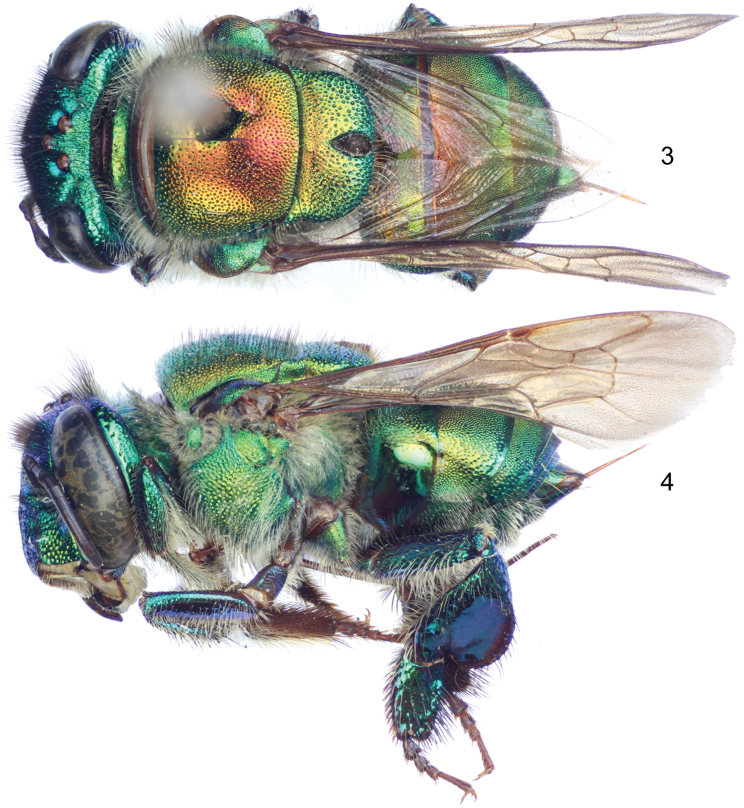
*Euglossa embera* sp. n., female paratype. **3** Dorsal habitus **4** Lateral habitus.

**Figures 5–13. F3:**
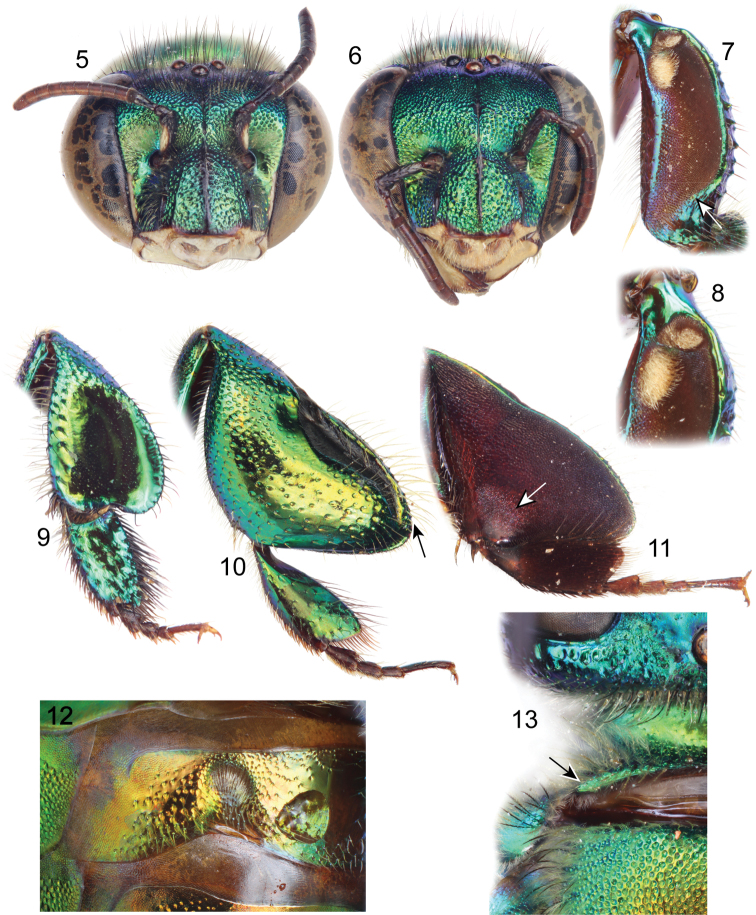
*Euglossa embera* sp. n. **5** Facial aspect of male holotype **6** Facial aspect of female paratype **7** Outer surface of male mesotibia (arrow pointing to oblique truncation of velvety area) **8** Mesotibial tufts of male **9** Outer view of female metatibia and metatarsus **10** Outer view of male metatibia and metatarsus (arrow pointing to distal-most extreme of organ slit) **11**Inner view of male metatibia and metatarsus (arrow pointing to circular depression) **12** Section of male second metasomal sternum **13** Dorsal view of pronotal dorso-lateral angle (arrow) of male.

Labiomaxillary complex in repose surpassing tip of metasoma by about one metasomal tergum length in the male (Figs 1–2), slightly shorter than metasoma in the female (Figs 3–4); both sexes with integument coloration light blue-green in the head, metasoma and mesosoma green with strong golden-bronzy coloration on dorsal areas as well as on metasomal sterna (Figs 1–4), male paraocular ivory marks narrow, not noticeably widened on lower sections, in most specimens not reaching epistomal sulcus (Figs 2, 5); male mandible tridentate, middle tooth reduced; female mesoscutellar tuft tear-drop shaped, occupying two thirds of mesoscutellar length (Fig. 3); female metabasitarsus trapezoidal with narrower straight distal margin, anterior and posterior margins convex (Fig. 9); metasomal terga in both sexes with dense punctures, becoming slightly shallower towards posterior margin; male mesotibia as follows: michrotrichia (velvety area) with anterior margin noticeably sparser, posterior margin obliquely truncate distally (Fig. 7), anterior mesotibial tuft ellipsoidal, proximal margin concave, and posterior mesotibial tuft elongated antero-posteriorly (Fig. 8); male metatibial shape scalene obtuse triangular, metatibial organ slit of male metatibia with basal section tear-drop shaped, distal section narrow and noticeably elongated, only separated from tibial ventral margin by less than the organ’s distal section maximum width (Fig. 10), inner surface with notorious circular depression near metabasitarsal joint (Fig. 11); male second metasomal sternum with two notorious omega-like integumental depressions (Fig. 12); male terminalia features as follows: eighth metasomal sternum with posterior section triangular (Fig. 15); dorsal process of gonocoxite thumb-like, about as long as broad (Fig. 18); lateral area of gonostylar process of gonocoxite projected as a short, broad prong; lateral section of gonostylus large, spoon-like, covered with dense, simple, long setae (Fig. 17). See also Table 1.

#### Description.

♂: *Structure* (all measurements in millimeters and based on 4 individuals). Total body length 11.62 (11.33–12.02); labiomaxillary complex in repose surpassing tip of metasoma by about one metasomal tergum length (Figs 1–2). Head length 2.54 (2.44–2.59), width 4.31 (4.21–4.43); upper interorbital distance 2.08 (2.04–2.15); lower interorbital distance 2.09 (2.07–2.15); upper clypeal width 1.13 (1.11–1.19); lower clypeal width 1.97 (1.93–2.00); clypeal protuberance 0.87 (0.81–0.93); medial clypeal ridge well developed, wide and blunt, paramedial clypeal ridges diagonal, well developed, sharp along their lower two thirds, obscured by punctation on upper third; labrum wider than long, length 1.09 (1.04–1.15), width 1.19 (1.15–1.22); medial labral ridge sharp; paramedial labral ridges blunt, oblique, running along length of labral windows; labral windows ovoid, occupying proximal two thirds of labrum; interocellar distance 0.34 (0.30–0.37); ocellocular distance 0.61 (0.59–0.65); first flagellomere shorter [0.37 (0.35–0.37)] than second and third flagellomeres combined [0.42 (0.37–0.44)]; length of malar area 0.19 (0.17–0.20). Mandible tridentate, middle tooth reduced, adjacent to inner margin of outer tooth. Pronotal dorso-lateral angle obliquely truncate (truncation appearing subtle, but noticeable), thicker (along the truncate edge) than remainder of posterior pronotal marginal ridge; intertegular distance 3.17 (3.04–3.33); mesoscutal length 2.61 (2.59–2.63); mesoscutellar length 1.19 (1.11–1.26); mesuscutum with no noticeable concavity on mesial area (at most with a thin linear shallow depression on posterior half); posterior margin of mesoscutellum evenly convex, convexity rather blunt on meso-posterior section (Fig. 1); mesotibial length 2.05 (2.00–2.07), mesotibial spur present; mesobasitarsal length 1.85 (1.78–1.93), width 0.71 (0.67–0.74) (as measured at proximal posterior keel), posterior keel projected in a slightly obtuse angle; metatibial shape triangular (scalene obtuse triangular), anterior margin noticeably convex on outer view (Figs 10-11), metatibial anterior margin length 3.11 (2.96–3.26), ventral margin length 2.43 (2.30–2.52), postero-dorsal margin length 4.28 (4.15–4.37), maximum metatibial thickness 1.10 (1.04–1.19); metatibial organ slit narrow, basal section teardrop shaped, anteriorly acute, length 0.67 (0.59–0.81), distal section spur shaped, elongated distally, separated from ventral margin by less than its maximum width, maximum width occupying about one-fourth of metatibial outer surface width (Fig. 10), metatibial inner surface with a notorious circular depression adjacent to joint with metabasitarsus (Fig. 11), metabasitarsal length 2.15 (2.07–2.22), mid-width 0.83 (0.81–0.89); metabasitarsal ventral margin oblique (Fig. 10). Forewing length 8.80 (8.67–8.96); jugal comb with 13–14 blades; hind wing with 16–20 hamuli. Maximum metasomal width 4.19 (4.07–4.30); second metasomal sternum with two shallow omega shaped depressions, lined with setae, located on concave areas of sinuate margin (Fig. 12).

*Coloration*. Head light blue-green with golden-bronzy iridescence specially on paraocular areas, antennal depressions and preoccipital area; clypeal medial ridge dark brown; paraocular ivory marks thin but well developed, slightly wider below, in most specimens not reaching epistomal sulcus (separated from it by about their width); lower lateral parts of clypeus, labrum, malar area, and mandibles (except teeth) ivory; labral windows amber-translucent; antennal scape with ivory spot covering all lateral surface and part of anterior surface, scape otherwise dark brown as remainder of antenna (Fig. 5). Pronotum green, blue-purple lights on lower ventral areas, golden-bronzy iridescence all over; mesoscutum, mesoscutellum and tegula green with strong golden-bronzy iridescence, dominant (obscuring green coloration) on mesoscutum and mesoscutellum (Figs 1–2); mesepisternum green with golden-bronzy iridescence specially on lateral areas (not as marked as on mesoscutum), preomaular area with brown-brassy spot on upper lateral area (Fig. 15); metepisternum and propodeum concolor with lateral areas of mesepisternum plus some blue-green coloration on areas close to leg joints; legs mainly bottle green on outer surface (except all tarsomeres beyond basitarsa) with moderate golden-bronzy iridescence, inner surface of all podites and entire tarsomeres beyond basitarsi brown-brassy, blue-purple lights on outer-anterior margins of most podites, specially notorious on mesofemur and mesotibia (Figs 2, 7); wings glossy hyaline with brown veins (Figs 1–2). Metasomal terga green with strong golden-bronzy iridescence in a gradient, strong anteriorly (fully bronzy) to weaker posteriorly (green-golden-bronzy) (Fig. 1); sterna with same colors and pattern as terga.

*Sculpturing*. Face densely areolate-punctate, areole-puncture size around one third of median ocellar diameter on clypeal disc, one eighth on frons (frontal fringe), and somewhere in between in other areas, paraocular groove between paraocular marks and torulus smooth (Fig. 5), gena with areole-punctures comparable in size to those of clypeal disc, well marked above, shallow on lower areas. Mesoscutum with round punctures about one fifth of median ocellar diameter, dense (separated by less than a puncture diameter) on most areas, becoming slightly sparser along median mesoscutal line (separated by one to two puncture diameters), where smaller punctures (about one fourth of a regular puncture size) are intermixed sparsely; mesoscutellum with punctation as on mesal areas of mesoscutum (sparse punctures intermixed with smaller punctures), punctures becoming denser (contiguous) and bigger (at least double in size) on posterior area along mesoscutal margin (Fig. 1); mesepisternal lateral-facing surface with dense punctures on upper areas as big as punctures on frons, becoming slightly bigger and sparser towards lower areas (separated by one or more puncture diameters on ventral areas); preomaular area with punctation as a continuation of lateral-facing area of mesepisternum, except for impunctate brown-brassy spot; metatibial outer surface with punctures comparable in size to those on posterior margin of mesoscutellum, relatively dense (separated by less than one puncture diameter) on upper half, sparser (separated by two to three puncture diameters) on lower half, smooth (impunctate) on small depression contiguous to organ slit (Fig. 10). Dorsal surface of posterior half of first metasomal tergum and second through fifth terga with dense punctures, around half the size of regular mesoscutal punctures, becoming slightly shallower towards posterior margin, anterior half of first tergum, lateral sections of second through fifth terga, and entire surface of sixth and seventh terga with similar pattern but punctures as big as those on posterior margin of mesoscutellum (Fig. 1); metasomal sterna with relatively dense punctation (punctures of a varied size, but most comparable to those on mesepisternum), leaving large semicircular smooth areas mesally on every sternum.

*Vestiture*. Frontal fringe with dense setae of two natures, some brown, very minutely branched, straight, as long as two mid-ocellar diameters, the others, amber-golden, with noticeable but short branches, shorter than the brown setae; remainder of the face (except as noted hereafter) with scattered amber-golden setae (as the ones on frontal fringe), shorter on most areas, and noticeably plumose on antennal depressions; posterior section of vertex and mid-ocellar area with long curved brown setae; gena with dense, light, plumose setae, increasing in size towards lower genal section; antenna with scattered amber golden setae (Fig. 5). Mesoscutum and mesoscutellum densely setose, majority of setae amber-golden with few intermixed brown setae (these last notorious on anterolateral corners of mesoscutum) (Figs 1–2, 13); lateral-facing surface of mesepisternum, metepisternum and propodeum with, dense, pale, plumose setae as long as those on lower gena, some brown setae interspaced on pronotal lobe; preomaular area with setae as those on lateral-facing mesepisternal areas, except bare on preomaular spot and contiguous smooth area (Fig. 2); outer surface of all legs with light yellowish setae, moderately dense and short in most areas except as follows: dense, long (as long as those on lower gena) and plumose on posterior surface of foreleg, dense and erect downwards on anterior surface of mesotibia (Fig. 7), dense and appressed on mesobasitarsus, long (as long as those on vertex) and arranged in a fringe on distal half of postero-dorsal margin of metatibia, other leg setal features as follows: inner surface of all basitarsi with dense, hirsute, brown-amber setae, chemical gathering tufts on second through fourth protarsomeres with dense, brown-amber, moderately long, setae, microtrichia on outer mesotibial surface (velvety area) composed of dense, fulvous, simple, minute setae, anterior margin of velvety area noticeably sparser, distal third of posterior margin diagonally truncate (Fig. 7), anterior mesotibial tuft ellipsoidal with proximal margin concave, composed of dense, fulvous, minutely plumose setae, posterior tuft sitting on a deep cavity elongated antero-posteriorly, with a distinctive semicircular setose patch on posterior half, anterior inner margin of the cavity covered with a fringe of setae, all setae fulvous (Fig. 8); metatibial organ slit closed with dark brown setae (Fig. 10); inner metatibial depression devoid of setae (Fig. 11). Anterolateral corners of first tergum, with moderately dense, amber-golden, simple setae as long as those on mesoscutum, lateral areas of all terga and posterior margin of seventh tergum with similar setae but rather pale, dorsum of posterior half of first tergum and second through sixth terga with dense, appressed, grayish, minute setae, intermixed with scattered, sturdy, brown, short setae appressed, similar setae but appearing simple, shorter and appressed, on lateral margins of remainder terga, as well as posterior half of fifth tergum and entire surface of sixth to seventh terga; posterior dorsal half of first tergum through anterior half of fifth tergum with dense, dusky, appressed short setae, intermixed with some scattered, darker, longer setae (Figs 1, 2); metasomal sterna with moderately dense setae on punctate areas; integumental omega-like depressions on second sternum lined with amber, appressed, simple setae (Fig. 12).

**Figures 14–20.**
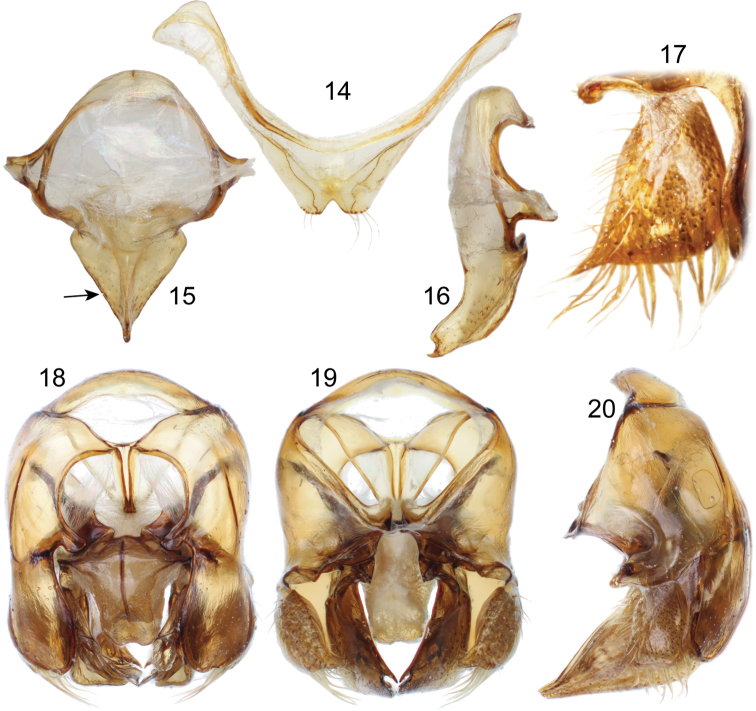
Male genitalic features of *Euglossa embera* sp. n. **14** Seventh metasomal sternum, ventral aspect **15** Eighth metasomal sternum, ventral aspect (arrow pointing to straight lateral margin) **16** Eighth metasomal sternum, lateral aspect **17** lateral section of gonostylus **18** Genitalic capsule, dorsal aspect **19** Genitalic capsule, ventral aspect **20** Genitalic capsule, lateral aspect.

*Terminalia*. Seventh metasomal sternum with posterior disc margin deeply emarginated mesally, bearing a row of scattered setae (Fig. 14). Eighth metasomal sternum with posterior section triangular (lateral margins straight) on dorsal or ventral view, covered with scattered, minute setae (Fig. 15). Dorsal process of gonocoxite thumb-like, about as long as broad, basal incision broadly concave (Fig. 18); lateral area of gonostylar process of gonocoxite projected as a short, broad prong; lateral section of gonostylus large, spoon-like, ventral lobe with scattered, short, simple setae on outer surface, inner concave surface covered with dense, simple, long setae (Fig. 17).

♀: *Structure* (all measurements in millimeters). Total body length 9.56; labiomaxillary complex in repose short of metasomal tip by less than one metasomal segment length (Fig. 4). Head length 2.48; head width 4.22; upper interorbital distance 2.11; lower interorbital distance 2.07; upper clypeal width 1.11; lower clypeal width 1.95; clypeal protuberance 0.74; medial clypeal ridge as in male, paramedial ridges weak, almost completely obscured by punctuation, labral ridges as in male, labral windows occupying about four fifths of labral length; labrum rectangular, wider than long, length 1.00, width 1.11; anterior edge of labrum arched outwards; interocellar distance 0.37; ocellocular distance 0.59; length of first flagellomere (0.37) shorter to combined length of second and third flagellomeres (0.41); length of malar area 0.09. Mandible tridentate. Pronotal lateral angle mainly as in male, but not so noticeably thicker than remainder of pronotal posterior ridge; intertegular distance 3.26; mesoscutal length 2.52; mesoscutellar length 1.26; posterior border of mesoscutellum as in male (Fig. 3); mesotibial length 2.00; mesobasitarsal length 1.63, maximum width 0.59; metatibia triangular (scalene right triangular) (Fig. 9), metatibial anterior margin length 2.81; metatibial ventral margin length 1.63; metatibial posterodorsal margin length 3.19; metabasitarsus trapezoidal with narrower straight distal margin, anterior and posterior margins convex (Fig. 9), length 1.70, maximum width 0.89. Forewing length 8.00; hind wing with 18 hamuli. Maximum metasomal width 4.30.

*Coloration*. As described for male (Figs 3–4). Paraocular marks, antennal scape spot, and preomaular spot absent (Fig. 6).

*Sculpturing*. As described for male except no differentiation on preomaular area (preomaular spot absent); mesoscutellum with slightly denser punctation (Fig. 3).

*Vestiture*. As described for male (some setal features on protarsi, meso- and metatibia exclusive of male) except as follows: Mesoscutellar tuft tear-drop shaped, occupying about two thirds of mid mesoscutellar length, composed of dense, dark, erect, multibranched (branches minute) setae (Fig. 3). Foreleg with slightly shorter setae on posterior surface as compared to male (Fig. 6); mesotibial posterior margin with some scattered, dark, sturdy short setae; metatibial corbicula surrounded for the most part by setae as in other leg areas, except by some scattered, dark, sturdy, curved setae (Fig. 9).

#### Etymology.

The specific epithet is a reference to the Emberá, an indigenous people inhabiting the Pacific lowlands of Colombia.

**Table 1.** Summary of useful male features for the species included in *Glossurella* as here restricted.

	*Euglossa prasina*	*Euglossa bursigera*	*Euglossa embera*	*Euglossa augaspis*	*Euglossa adiastola*a
**Shape of metatibia**	Trapezoidal (posterior angle orthogonal), inflated	Triangular (posterior angle acute)	Triangular (posterior angle acute)	Triangular (posterior angle acute)	Triangular (posterior angle acute)
**Shape of scape**	Inflated, club-like	Cylindrical	Cylindrical	Cylindrical	Cylindrical
**Length of labiomaxillary complex**	Reaching but not surpassing tip of metasoma	Reaching or barely surpassing tip of metasoma	Clearly surpassing tip of metasoma (by about half a metasomal segment length)	Reaching or barely surpassing tip of metasoma	Reaching or barely surpassing tip of metasoma
**Malar length**	About ⅓ width of mid-flagellomeres	About ½ width of mid-flagellomeres	About ¾ width of mid-flagellomeres	About ½ width of mid-flagellomeres	About ⅓ width of mid-flagellomeres
**Separation of tip of metatibial organ slit from ventral margin of metatibia**	As long as total length of organ slit (or slightly longer)	Slightly over maximum width of organ slit	Noticeably less than maximum width of organ slit	About 1½ maximum width of organ slit	About 1½ maximum width of organ slit
**Mid-mandibular tooth**	Well differentiated from outer tooth	Minute, adjacent to outer tooth	Minute, adjacent to outer tooth	Minute, adjacent to outer tooth	Well differentiated from outer tooth
**Known distribution**	Amazon Basin	Central America	Pacific lowlands of Colombia	Amazon Basin	Coastal areas of northern Atlantic Forest in Brazil

### Euglossa (Glossurella) adiastola sp. n.

urn:lsid:zoobank.org:act:72E3F6A4-847D-40D3-B78E-44AB469DED67

http://species-id.net/wiki/Euglossa_adiastola

Figs 21 28

#### Holotype.

♂, labeled: “Euglossina da; Hiléia Baiana; REBIO C. Veado; 18289 – 52751 // Pinheiros ES; BRASIL 09/02/2010; A. Nemésio”. The holotype is deposited in UFMG.

#### Paratypes.

15♂♂: labeled as follows: data as holotype (1♂) except date “07/02/2010” and lacking first label; data as holotype (4♂) except individual file numbers “18240-52566”, “18244-52581”, “18244-52583” and “18270-52685”; “Euglossina da; Hiléia Baiana; REBIO C. Grande; 18059 – 51976 // Conceição Barra ES; BRASIL 02/02/2010; A. Nemésio” (1♂); “Euglossina da; Hiléia Baiana; Res. Nat. Vale; 17096 – 48063 // Linhares ES; BRASIL 10/12/2009; A. Nemésio” (1♂); “Euglossina da; Hiléia Baiana; RPPN Duas Barras; 18680 – 53528 // Sta Maria do Salto MG; BRASIL 12/02/2009; A. Nemésio” (1♂); “Euglossina da; Hiléia Baiana; PN Monte Pascoal; 16456 – 46333 // Porto Seguro BA; BRASIL 05/10/2009; A. Nemésio” (1♂); “Euglossina da; Hiléia Baiana; PN Descobrimento; 16492 – 46415 // Prado BA; BRASIL 07/10/2009; A. Nemésio” (1♂); “Euglossina da; Hiléia Baiana; Res. Ecol. Michelin; 16828 – 47244 // Igrapiúna BA; BRASIL 27/11/2009; A. Nemésio” (1♂); “Euglossina da; Hiléia Baiana; P. E. S. Conduru; 17990 – 51710 // Uruçuca BA; BRASIL 30/01/2010; A. Nemésio” (1♂); “Euglossina da; Hiléia Baiana; RPPN Serra Bonita; 17807 – 51142 // Camacan BA; BRASIL 24/01/2010; A. Nemésio” (1♂); “Euglossina da; Hiléia Baiana; Campus UESC; 18331 – 52871 // Ilhéus BA; BRASIL 20/02/2010; A. Nemésio” (1♂); “Ilhéus BA; BRASIL 27/07/2010; A. Nemésio” (1♂). Paratypes are deposited in UFMG, except the first and the last ones are deposited in SEMC.

#### Diagnosis.

**Figures 21–22.**
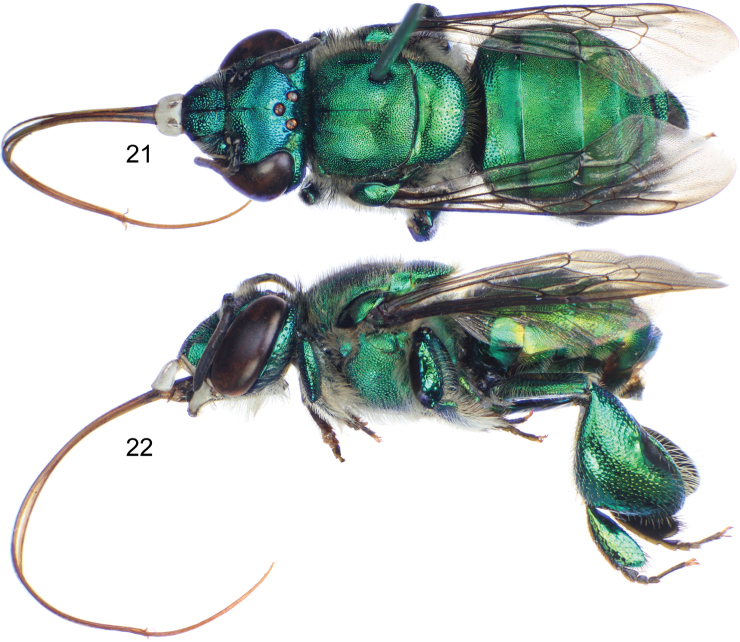
*Euglossa adiastola* sp. n., male holotype. **21** Dorsal habitus **22** Lateral habitus.

**Figures 23–28.**
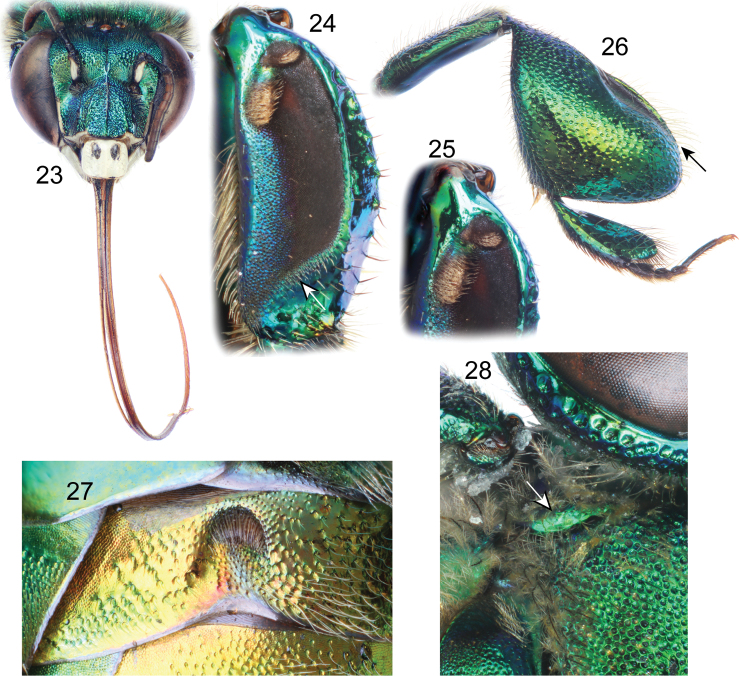
*Euglossa adiastola* sp. n. male holotype. **23** Facial aspect **24** Outer surface of mesotibia (arrow pointing to oblique-concave truncation of velvety area) **25** Mesotibial tufts **26** Outer view of metatibia and metatarsus (arrow pointing to distal-most extreme of organ slit) **27** Section of second metasomal sternum **28** Dorsal view of pronotal dorso-lateral angle (arrow).

Labiomaxillary complex in repose reaching tip of metasoma (estimation) (Figs 21–22); integument coloration light blue-green in the head, uniformly bottle green on metasoma and mesosoma, moderate golden-bronzy iridescence all over (strong on metasomal sterna) (Figs 21–22), paraocular ivory marks narrow, not noticeably widened on lower sections, reaching epistomal sulcus (Fig. 23); mandible tridentate, middle tooth well developed; mesotibial microtrichia (velvety area) with anterior margin noticeably sparser, posterior margin obliquely truncate distally (concave margin in oblique section) (Fig. 24), anterior mesotibial tuft ellipsoidal, proximal margin concave, posterior mesotibial tuft elongated antero-posteriorly (Fig. 25); metatibial shape scalene triangular, organ slit with basal section tear-drop shaped, distal section very narrow separated from tibial ventral margin by more than its maximum width (Fig. 26); male second metasomal sternum with two notorious omega-like integumental depressions (Fig. 27); male terminalia features as described for *Euglossa embera*. See also Table 1.

#### Description.

♂: Structure (all measurements in millimeters and based on the holotype). Total body length 12.81; labiomaxillary complex in repose reaching tip of metasoma or even exceeding it by 1–2 mm in some specimens (Figs 21–22). Head length 2.78, width 4.50; upper interorbital distance 2.22; lower interorbital distance 2.15; upper clypeal width 1.19; lower clypeal width 1.96; clypeal protuberance 0.81; clypeal ridges as described for *Euglossa embera*; labrum wider than long, length 0.96, width 1.15; medial labral ridge sharp; paramedial labral ridges blunt, oblique, running slightly beyond length of labral windows; labral windows ovoid, occupying proximal half of labrum; interocellar distance 0.44; ocellocular distance 0.59; first flagellomere slightly shorter (0.41) than second and third flagellomeres combined (0.44); length of malar area 0.19. Mandible tridentate, middle tooth well developed and differentiated from outer tooth. Pronotal dorso-lateral angle obliquely truncate, noticeably thicker than remainder of posterior pronotal marginal ridge (Fig. 28); intertegular distance 3.56; mesoscutal length 2.89; mesoscutellar length 1.26; mesoscutellum as described for *Euglossa embera* (Fig. 21); mesotibial length 2.15, mesotibial spur present; mesobasitarsal length 2.00, width 0.74 (as measured at proximal posterior keel), posterior keel projected in a obtuse angle; metatibial shape triangular (scalene triangular), anterior margin rather straight on outer view (Fig. 26), metatibial anterior margin length 2.96, ventral margin length 2.74, postero-dorsal margin length 4.37, maximum metatibial thickness 1.30; metatibial organ slit narrow (narrower than in *Euglossa embera*), basal section teardrop shaped, anteriorly acute, length 0.59, distal section spur shaped, separated from ventral margin by more than its maximum width, maximum width occupying about one-fifth of metatibial outer surface width (Fig. 26), metatibial inner surface as in *Euglossa embera*, metabasitarsal length 2.30, mid-width 0.81; metabasitarsal ventral margin oblique (Fig. 26). Forewing length 9.48; jugal comb with 13–14 blades; hind wing with 17–18 hamuli. Maximum metasomal width 4.67; second metasomal sternum with two depressions as described for *Euglossa embera* (Fig. 27).

*Coloration*. Head as described for *Euglossa embera*, except as follows: golden-bronzy coloration not as strong, paraocular ivory marks reaching epistomal sulcus, ivory spot on antennal scape extended on frontal surface (covering most of it) (Fig. 23). Mesosoma uniformly bottle green with golden-bronzy hue (Figs 21–22); legs as described for *Euglossa embera* (Figs 22, 24–26); wings glossy hyaline with dark brown veins (Figs 21–22). Metasomal terga bottle green with golden-bronzy iridescence (accentuated on lateral areas) (Figs 21–22); sterna as in *Euglossa embera*.

*Sculpturing*. In general as described for *Euglossa embera*, except as follows: punctation along median mesoscutal line not as sparse, although sparser than elsewhere; metatibial outer surface with denser punctation, area along ventral margin with punctures separated by one to two puncture diameters (Fig. 26).

*Vestiture*. In general as described for *Euglossa embera*, except as follows: setae on meso- and metasoma, evenly fulvous, mesoscutum and mesoscutellum with a noticeable number of intermixed brown sturdy setae; anterior section of velvety area on mesotibia, sparser than in *Euglossa embera*, distal third of posterior margin of velvety area rather concave (Fig. 24).

*Terminalia*. As described for *Euglossa embera*.

♀: Unknown.

#### Etymology.

The specific epithet is based on the Greek word *adiastolos*, meaning “confused” or “not separated”, as a reference to the confusion between this species and *Euglossa augaspis*.

## Discussion

The two new species described here are unequivocally related to *Euglossa bursigera* Moure, *Euglossa augaspis* Dressler, and *Euglossa prasina* Dressler. The males of all of these species have characteristically tridentate mandibles and share a similar habitus, integumental sculpturing, and genitalic features. It is easy to separate *Euglossa prasina* from those species by its distinctive metatibial shape (trapezoidal), and rather enlarged scape (see also Table 1). Before the addition of the species here described, it was relatively easy to distinguish *Euglossa bursigera* and *Euglossa augaspis* based on their distribution because the former is found in Central America (Moure 1970) while the second is found in the Amazon Basin (Dressler 1982b). Both species share a good amount of morphological similarity, and this is also the case for *Euglossa embera* and *Euglossa adiastola*. *Euglossa embera* is closer to *Euglossa bursigera*, and although not from Central America, the type material is from a biogeographically related region, the Pacific lowlands of Colombia; *Euglossa adiastola* on the other hand is more akin to *Euglossa augaspis*.

The four males known for *Euglossa embera* are remarkably uniform in their morphology which is not surprising given that all of them come from the same locality. This species can be differentiated from *Euglossa bursigera* by the noticeably longer labiomaxillary complex of the male (clearly exceeding the metasomal tip while in repose), which does not surpass or barely surpasses the metasomal tip in *Euglossa bursigera*; the slightly longer malar area in the male (length comparable to width of the mid-flagellomeres), which is much narrower than the mid-flagellomeres width in *Euglossa bursigera*; and the posteriorly elongate distal section of the metatibial organ slit (separated from ventral metatibial margin by less than its maximum width), which in *Euglossa bursigera* is separated from the ventral metatibial margin by more than the slit’s maximum width (see Table 1). Coloration could also be used to differentiate these two species, although Moure (1970) described a subspecies of *Euglossa bursigera* (*Euglossa bursigera cupreicolor*), based on the dominant bronzy-reddish coloration of specimens found principally (but not exclusively) in the Pacific slope of Costa Rica. Most of the Panamanian specimens of *Euglossa bursigera* are, however, predominantly green. Collection of specimens in the contact areas of both *Euglossa bursigera* and *Euglossa embera* will help clarify the color variation in *Euglossa bursigera*.

Dressler (1982b), when describing *Euglossa augaspis*, addressed the close morphological similarity of this species with *Euglossa bursigera* from which he distinguished it by its distinctively smaller size and denser abdominal punctation. *Euglossa adiastola*, although definitely closer to *Euglossa augaspis*, is noticeably larger, even slightly larger than *Euglossa bursigera*. Besides size, *Euglossa adiastola* can be distinguished from *Euglossa augaspis* by the much thicker dorso-lateral angle of the prothorax and the well-developed middle tooth in the mandible.

*Euglossa adiastola* has a relatively wide distribution in the northern portion of the coastal Atlantic Forest. It has been listed, and may be found in entomological collections, as *Euglossa augaspis* from the states of Pernambuco (Milet-Pinheiro and Schlindwein 2005), Bahia (Nemésio 2009, 2011a), Minas Gerais (Nemésio 2012), and Espírito Santo (Bonilla-Gómez 1999; Nemésio 2011b).

The new species here presented together with *Euglossa bursigera*, *Euglossa augaspis*, and *Euglossa prasina* are assigned to the subgenus *Glossurella*. When Dressler (1982b) originally erected the subgenus, he included a variety of species that share some biological (nesting) and external morphological features. However, currently available phylogenetic information based both on external morphology (Hinojosa-Díaz 2010) and molecular data (Ramírez et al. 2010) indicate that the group as envisioned by Dressler (1982b) is not supported as monophyletic. Since the type species for *Glossurella* is *Euglossa bursigera* we tentatively restrict the use of this subgeneric name herein for those species allied to it. As so conceived, such a restricted *Glossurella* would encompass *Euglossa bursigera*, *Euglossa augaspis*, *Euglossa prasina*, *Euglossa embera*, and *Euglossa adiastola*. Species formerly included in *Glossurella* but not part of the complex allied to *Euglossa bursigera*, would then be regarded as *incertae sedis* in terms of their subgeneric placement within *Euglossa* and until such time as relationships are further resolved(e.g., Hinojosa-Díazand and Engel 2011b). Naturally, this is one of several classificatory options but is the one which offers the greatest nomenclatural stability for the moment.

## Supplementary Material

XML Treatment for Euglossa (Glossurella) embera

XML Treatment for Euglossa (Glossurella) adiastola
